# Real‐time dielectrophoretic signaling and image quantification methods for evaluating electrokinetic properties of nanoparticles

**DOI:** 10.1002/elps.201400500

**Published:** 2015-07-06

**Authors:** David J. Bakewell, Joe Bailey, David Holmes

**Affiliations:** ^1^ Department of Electrical Engineering and ElectronicsUniversity of LiverpoolLiverpoolUK; ^2^ London Centre for NanotechnologyUniversity College LondonLondonUK; ^3^ CoMPLEX: Centre for Mathematics and Physics in the Life Sciences and Experimental BiologyUniversity College LondonLondonUK; ^4^ Sphere Fluidics LtdBabraham Research CampusBabrahamCambridgeUK

**Keywords:** AC electrokinetics, Collection rate, Dielectrophoresis, Nanoparticle, Real‐time measurement

## Abstract

Real‐time image signaling and quantification methods are described that allow easy‐to‐use, fast extraction of the electrical properties of nanoparticles. Positive dielectrophoretic (pDEP) collection rate analysis enables the dielectric properties of very small samples of nanoparticles to be accurately quantified. Advancing earlier work involving dual‐cycle pulsed pDEP 1 collection experiments, we report the development of a statistical image quantification method that significantly advances the evaluation of nanoparticle dielectric properties. Compared with traditional methods that require information about the geometry of the electrode array to be entered for semiautomated quantification 2, the new statistical approach described does not require a priori knowledge of device geometry. The efficacy of the statistical method is experimentally demonstrated using 200 nm diameter latex nanospheres, suspended in low conductivity medium, that are attracted by pDEP onto planar castellated electrode arrays with 5‐micron‐sized features. The method is shown to yield estimates for the nanoparticle conductivity and surface conductance, $\sigma_{p}\;\;=\;\;25.8$ mS/m and $K_{S}\;\;=\;\;1.29$ nS, that concur closely with those obtained using traditional geometric methods previously reported 1. Consequently, the statistical method is accurate, fast, robust, supervisor‐free, and useful for determining nanoparticle electrokinetic parameters.

**Abbreviations**
DEP: dielectrophoresis
nDEP: negative DEP
pDEP: positive DEP
RoI: region of interest
RF: radio frequency

## 1 Introduction

**Figure 1:**
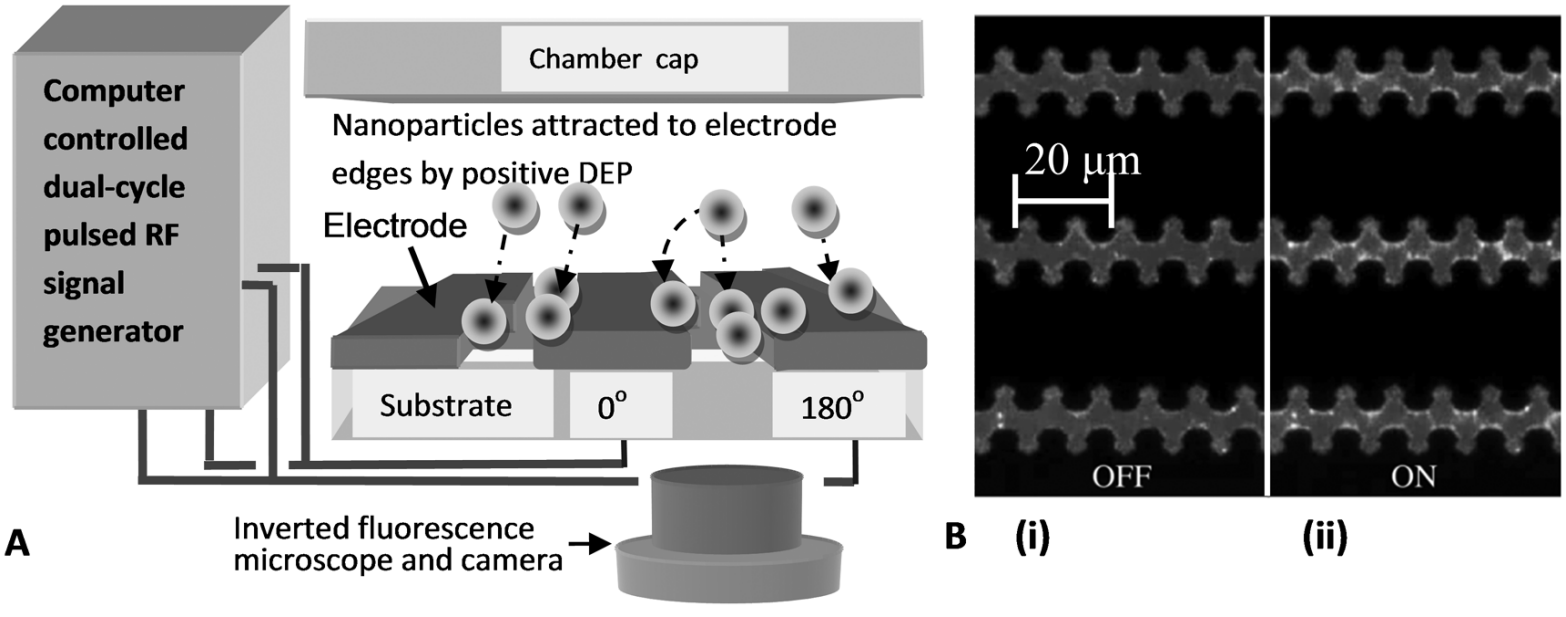
(A) Schematic of a typical experimental arrangement showing nanoparticles being attracted by pDEP onto microfabricated planar castellated electrodes, connected to a signal supply, as shown. The experiment is imaged using an inverted fluorescence microscope (not to scale, see text for details). (B) Typical partial‐frame images of fluorescent nanoparticles with pDEP (i) *off* and (ii) *on*. The dark regions are the castellated electrodes. During the “on” state fluorescently labelled nanoparticles collect at the electrode tips as an AC signal is applied.

The electrical properties of nanoparticles play a key role in determining their dispersive behavior in aqueous solution. Charge screening and mutual nanoparticle repulsion, for example, are influenced by co‐ and counterions associated with the electrical double layer. Nanoparticle electrical properties are in turn dependent on their intended application, for instance, biological labeling influences nanoparticle surface charge as well as other properties 3. An important electrokinetic laboratory technique for nanoparticle electrical characterization, requiring microliter suspension volumes or less, is positive dielectrophoretic (pDEP) collection rate measurement. Dielectrophoresis (DEP) is the translational movement of an electrically polarizable body, suspended in a suitable medium, by the action of a nonuniform electric field 4, 5. The dielectric properties of the body can be determined by measuring its movement under the influence of DEP, which is typically implemented by applying a low voltage radio frequency (RF) electrical signal to microfabricated electrodes immersed in an electrolyte of low conductivity, as shown in Fig. 1A. DEP is a powerful technique that has become popular in recent years due to the convenience with which it can be integrated into lab‐on‐chip platforms 6, 7, 8.

The dielectric properties of nanoparticles are evaluated by measuring the initial rate of their collection under the action of pDEP immediately after the force is switched on. The initial collection rate is approximately proportional to the small‐time averaged DEP force 5, 9, 10, 11, 12, 13, 14, 15, 16, 17, 18, 19, 20,

(1) F⃗ DEP (x̲,t)=2πr3ɛm Re {f CM [ω(t)]}∇⃗|E⃗(x̲)|2

where $\underline{x}$ is the spatial position, *r* is the spherical nanoparticle radius, $|\vec{E}|$ is the electric field magnitude (root of mean of square), ε_m_ is the medium permittivity,  Re {f CM [ω(t)]} is the real part of the Clausius–Mossotti function with angular frequency, ω(t)=2πf(t) that can be switched in time, *t*, and other symbols have been previously defined 5, 8. The real part of the Clausius–Mossotti function is abbreviated, for convenience, as  Re {f CM [ω(t)]}=f CM R and apart from being frequency dependent, f CM R is understood to be dependent on the respective permittivity and conductivity of the particle, ε_p_ and σ_p_, and medium, ε_m_ and σ_m_. Usually the DEP force is switched on for a limited time (up to a minute) then switched off. Repeating the procedure leads to pulsed DEP 1, 2, 17, 18, 19, 20, 21, 22, 23, 24, 25. As illustrated in Fig. 1B(i), during DEP “off,” nanoparticles diffuse randomly so that their distribution within a confined chamber volume becomes uniformly distributed. When pDEP is switched “on” (collection phase) the pDEP forces result in nanoparticles moving toward the electrode tips, as shown in Fig. 1B(ii). A single cycle typically comprises a *collection* phase (pDEP “on”) followed by a *release* phase (pDEP switched “off”). The label for the latter phase stems from the absence of pDEP that allows nanoparticles to diffuse and be “released” from the electrodes.

**Figure 2:**
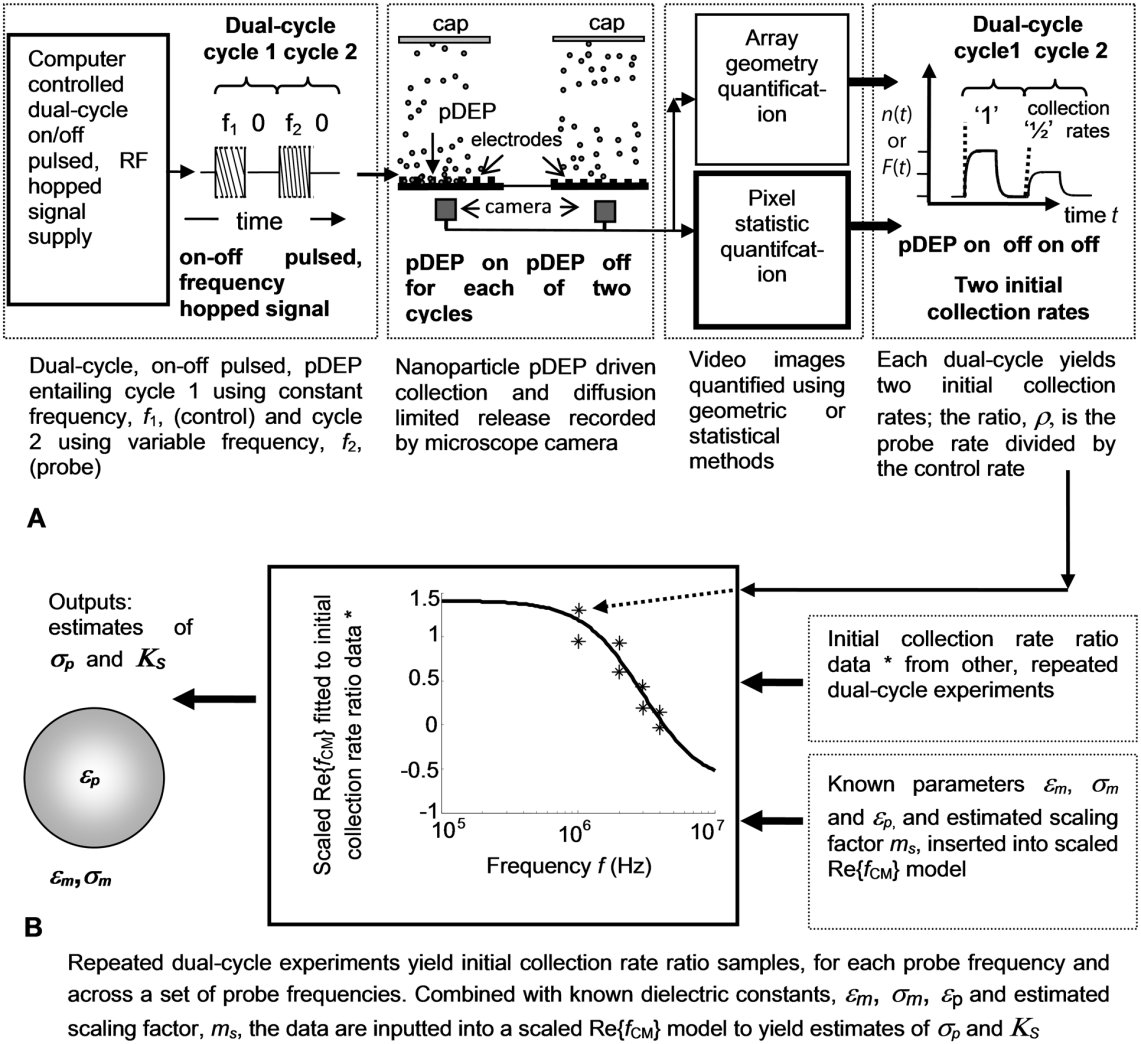
(A) Dual‐cycle, on–off pulsed, frequency hopped pDEP and fluorescence microscope image quantification using geometric or statistical methods—the latter which is the focus of this article. The dual‐cycle effectively generates a ratio of the probe nanoparticle number (or fluorescence) initial collection rate divided by the control, as shown. (B) Initial collection rate ratio data, spanning a range of frequencies, and in conjunction with other experimental parameters, enables fitting of the scaled Re{*f*_CM_} and evaluation of the nanoparticle conductivity and surface conductance.

Pulsed DEP has been used to characterize the electrical properties of cells and their constituents, for example DNA, ribonucleic acid, viruses, and colloidal bioparticles 1, 2, 4, 5, 6, 7, 8, 9, 10, 11, 12, 13, 14, 15, 16, 17, 18, 19, 20, 21, 22, 23, 24, 25, and in many investigations, initial collection rates were measured and fitted to f CM R. A newly developed, fast alternative to traditional methods for characterizing electrical properties, for example, cross‐over measurements 26, 27, 28, 29, 30, is dual‐cycle continuously pulsed DEP 1 shown by the scheme illustrated in Fig. 2A and B. Referring to Fig. 2A, the first cycle collection phase uses a constant frequency that acts as a *control*, that is, $f\left(t\right)\;\;=\;f_{0}$, and it was introduced in previous work 1 to reduce the amount of experimental variation or uncertainty. The second cycle collection phase uses a frequency that is made variable so it can *probe* the DEP response for a range of selected frequencies, that is, $f\left(t\right)\;\;=\;\;f_{i},\;\;i\;=\;1,\;2,\;3,\;...$ Consequently, each dual cycle is capable of yielding two initial collection rates, as shown in Fig. 2A.

The initial collection rate *ratio* for each *i*th probe frequency, ρ_i_, is the initial collection rate of the probe nanoparticle number, *n*(*t*), or fluorescence, *F*(*t*), divided by the initial collection rate of the control. It is expressed by Eq. (9) in 1 and it can be written as ρi=msf CM R(ωi) where ms=1/f CM R(ω0) and acts as a f CM R *magnitude scaling* factor. That is, ρ_i_ is equal to a scaled f CM R, as shown by 1 and Supporting Information Eq. (S1.2). Since typical values are $0.5\;\;<\;\;m_{s}\;\;<\;\;2$, then ρ_i_ is not strictly bound between −0.5 and 1. Figure 2B shows an example of initial collection rate ratio data generated by dual‐cycle pDEP collection and release experiments for a range of frequencies. As each experiment is repeated with a probe frequency increasing from a few hundred kilohertz up to tens of megahertz, the frequency‐dependent polarizability decreases; sufficiently higher probe frequencies result in negative DEP (nDEP). Importantly, the scaled f CM R involves six parameter values to yield a single value of the ratio, ρ_i_, that is, ε_m_, σ_m_, ε_p_, σ_p_, *m*_s_ and $\omega_{\;i}$. Consequently, an *inverse* process that assumes known values for ε_m_, σ_m_ and ε_p_, and fits a *set* of experimentally measured initial collection rate ratios with their corresponding frequencies, $\left\{\rho_{i}\right\}\;\;=\;\;\left\{\rho_{1},\;\rho_{2},\;..\right\}$ and $\left\{\omega_{\;i}\right\}\;\;=\;\;\left\{\omega_{1},\;\omega_{2},...\right\}$, can be used to jointly estimate *m*_s_ and σ_p_. Using the relation, $K_{S}\;\;=\;\;\sigma_{p}\;r/2$, the surface conductance can also be estimated, as illustrated in Fig. 2B.

Nanoparticle collection rate measurements mostly involve imaging with a fluorescence microscope and postprocessing the resultant videos using software developed by individual researchers 17, 21. Image processing reports include, for example, cells suspended in circular apertures and single feature structures 31, 32, 33, 34, 35, otherwise the literature lacks treatment concerning DEP image quantification, particularly for multiple, small feature, intricate electrode array designs. Conventionally, pDEP initial collection rates are measured by evaluating the initial rate of change of optical intensity (e.g. fluorescence) and quantified frame by frame. Evaluation of the 2D spatial mean intensity for each frame yields noisy and unsatisfactory collection time profiles so the S/N is improved by selecting intensities over designated 2D regions associated with pDEP, e.g. near electrode tips. To evaluate the intensity exclusively in these regions information about the array geometry is necessary so the conventional approach is called *geometric filtering*, as referred in Fig. 2A. Although computer‐based feature extraction methods have been developed, for example, for castellated arrays 2, extending the methods for more intricate designs involves cost in time, effort, and expertise.

The paper describes the development of a novel image quantification method for use in DEP experiments and demonstrates the utility of the method using planar castellated arrays. A range of dual‐cycle probe frequencies is used to experimentally determine values for *m*_s_, σ_p_, and *K*_S_ by measuring the collection rates at each frequency using the new approach of statistical filtering, referred to Fig. 2A, that offers an alternative to geometric filtering. The results concur closely with estimates of σ_p_ and *K*_S_ using conventional, geometric filtering. Consequently, ratios of pDEP collection rates, for example, dual‐cycle 1, can be rapidly quantified in real‐time and without needing supervisory input, thus, saving considerable resources and significantly advancing earlier work 1, 2, 5, 9, 10, 11, 12, 13, 14, 15, 16, 17, 18, 19, 20, 21, 22, 23, 24, 25.

## 2 Materials and methods

The fluorescence intensity at a given point, for low particle concentrations used in this work, is proportional to the concentration of nanoparticles 1, 2, 15, 16, 17. Importantly, the length scale of each pixel in each frame is of the same order as the nanoparticle diameter so that the light intensity measured by pixels for a single fluorescent nanoparticle will be distributed and dependent on the distance from the optical focal plane and other factors. Additionally, even if nanoparticles are uniformly distributed over frame‐size lengths during DEP “off,” there can be small‐scale, feature‐sized areas where the nanoparticle densities vary and this behavior is indicated by the distribution of frame pixel intensities.

### 2.1 Image processing using information about array geometry

Typically, there are two steps in the geometric filtering of periodic electrode array videos. The first step considers the geometry of the electrodes and involves extracting required information about the array geometry (e.g. the positions of the electrode edges) either manually [1, 15, 16, 17, 21] or by bespoke computer software, for example, feature recognition 2. The image is then partitioned and spatially averaged over the periodically repeating (10's–100's) unit cell of the electrode array. The second step uses the information from the first step and involves defining a region of interest (RoI) in each cell.

**Figure 3:**
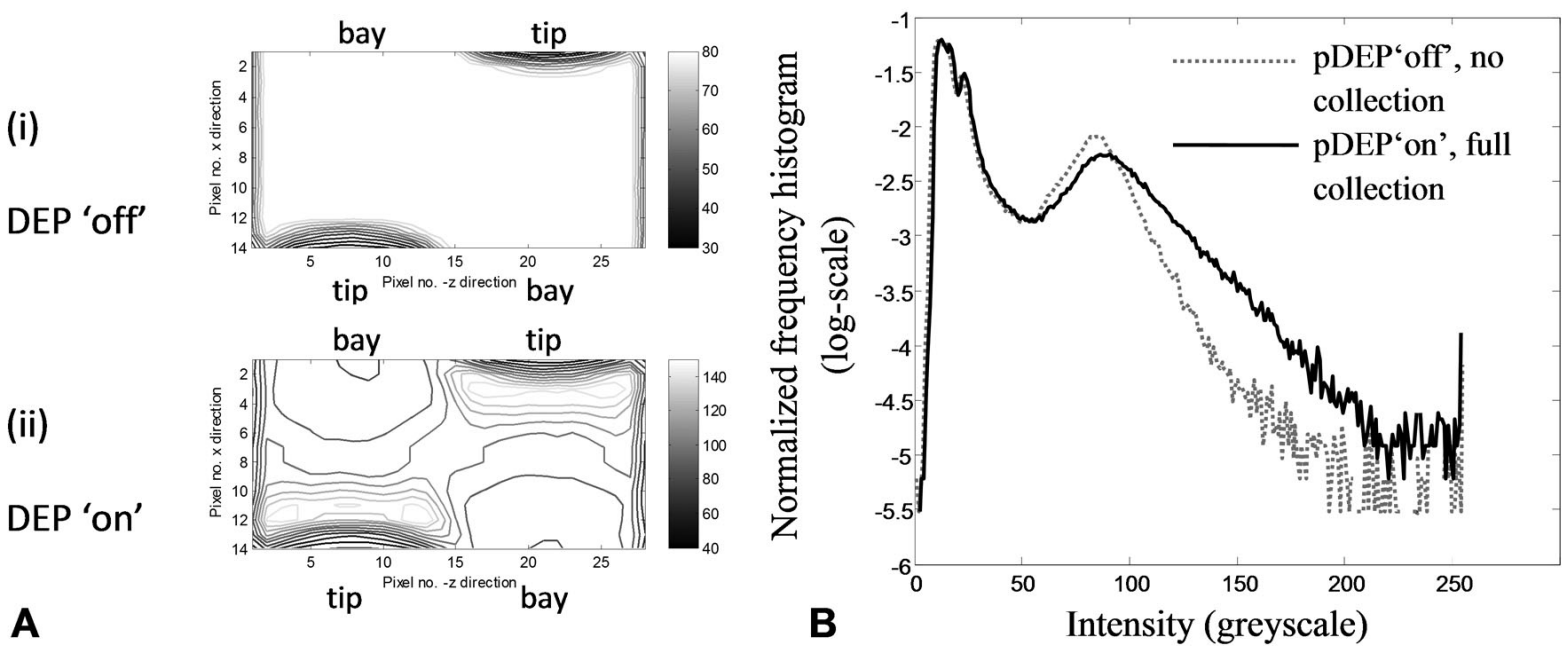
(A) Intensity contours (grayscale units) for the rectangular region of interest representing the fluorescence intensity measured over the interelectrode gap (castellated electrode tips and bay regions as labelled). (i) DEP “off”—the dark contours indicate the electrode edges and the lighter areas are due to scattered light that is uniformly distributed, as shown by the absence of contours. (ii) DEP “on”—light gray contours, with grayscale values well above scattered light, show pDEP‐driven nanoparticle collections near the electrode tips and across the interelectrode gap. On the other hand, nanoparticle depletion is indicated by darker contours associated with the bay regions. (B) Statistical filtering considers the pixel intensity distribution (normalized frequency histogram) for the entire frame. The gray dotted curve shows the distribution for the pDEP “off” state; the black full curve shows the distribution for the pDEP “on” state. By choosing appropriate statistics sensitive to the distribution spread or SD, the different off–on states can be detected without needing user input data.

For example, referring to Fig. 3A, the RoIs shown in (i) and (ii) extend across the interelectrode gap. The 2D spatial mean of pixel intensity for the RoI is evaluated for each frame of the video and the initial collection rates are calculated from the data. Geometric filtering offers a direct and intuitive approach to measurement of collection rates. Only pixels from each unit cell RoI contribute to the output, $\mu_{G}\left(t_{m}\right)\;$ where μ denotes 2D spatial *mean*, subscript “*G*” indicates that the RoI is the interelectrode *gap*, and *t*_m_ is discrete time in terms of frame number, *m*. The drawbacks of geometric filtering are the complexity of the image processing quantification and requirement for in‐house expertise needed to develop video processing software.

### 2.2 Image processing using statistical methods

An alternative to separating each image into its spatial components is to consider the distribution of pixel intensities. Typical pixel intensity distributions (normalized frequency histograms, made continuous by interpolation) for two frames are plotted in Fig. 3B. Both distributions exhibit two modes the left, “dark” mode for the very low intensities represent the regions above the electrodes, and the right mode for the moderately low intensities represents the fluorescence from the nanoparticle suspension. Comparing DEP “on” (black line) distribution in Fig. 3B with the DEP “off” (gray) distribution distinguishes two important features. First, pixel values near the right, “brighter”, mode peak that indicate moderately low nanoparticle concentration *decrease* with the onset of pDEP. Second, pixel values in the distribution tails, indicating high concentration, *increase* markedly with pDEP and are consistent with pDEP forming clusters of nanoparticles. Thus, the tail of the pixel distribution is a sensitive indicator of pDEP collection. The statistical interpretation concurs with experimental observations in terms of array geometry. For example, nanoparticle enhancement occurs across the interelectrode gap due to pDEP, particularly near the electrode tips, as shown by the light gray contours in Fig. 3A(ii). Depletion of nanoparticles occurs in regions where there is no pDEP, indicated by the dark contours associated with the electrode bay regions.

The prominence of the left mode in Fig. 3B explains why the 2D spatial mean intensity for each frame yields noisy, unsatisfactory collection time profiles, thus showing a need for filtering. A statistical measure that is sensitive to the shift in the high pixel intensity tail of the distribution, due to pulsed pDEP, is the SD of the frame pixel distribution. The expression for an asymptotically unbiased estimator of the SD 36, applicable for a large number of pixels in the frame, is as follows:

(2) $$\sigma_{F}\left(t_{m}\right)=\sqrt{\bar{I_{g}^{2}}\left(t_{m}\right)\;-\;\;{{\bar{I}}_{g}}^{2}\left(t_{m}\right)}$$

where the second moment, $\bar{I_{g}^{2}}\left(t_{m}\right)=\frac{1}{n_{p}}\sum_{k\;=\;1}^{n_{p}}I_{g}^{2}\left(k,\;t_{m}\right)$, and similarly for the first moment, or mean. In Eq. (2), *n*_p_ is the number of pixels in the frame, σ denotes SD, subscript “*F*” denotes the entire *frame*, and the pixel intensity, *I* , adopts the subscript “g” to denote *greyscale* units.. The moments of the pixel intensities, $I_{g}\left(k,\;t_{m}\right)$, are *independent* of any geometric features that the video frame may represent. For example, the index, *k*, is only involved in summing the pixel intensities in Eq. (2), it does *not* dependent on, or refer to, any spatial array coordinate. Consequently, Eq. (2) may be straightforwardly applied to a variety of electrode geometries and the scaling of the demands is simply dependent on *n*_p_. Thus, the *statistical* filtering method is more amenable to intricate, extensive array designs than geometric filtering.

### 2.3 Experimental methods

Referring to Figs. 1A and 2A, an arbitrary function generator (Tektronix AFG 3022B, OR, USA) was used to provide low voltage, square wave enveloped, sinusoidal signals applied to the electrodes with fixed control frequency (0.7 MHz) and variable probe frequency (100 kHz–10 MHz). Equal dual‐cycle periods, T=17s, comprising 7 s signal on, 10 s signal off were applied and the ground‐to‐peak voltage, during the DEP collection phase, was 1.0 V for all experiments. Each experiment was video recorded with frame rate 10 frames/s. Collections for the first cycle were transient whereas the four remaining cycles exhibited cyclic steady state 1, 21. Pulse duration, amplitude and applied frequencies were controlled by custom software written in LabVIEW™ 2011 (National Instruments, Austin, TX, USA).

Castellated geometry interdigitated microelectrode arrays, shown in Fig. 1B, with edge sizes of 5 microns were fabricated using lift‐off techniques. A 100 nm thick layer of platinum was patterned on 500 μm pyrex wafers and the arrays were cut from the wafer and mounted on a printed circuit board to allow robust electrical connection to the signal generator. A 3 mm internal diameter sample reservoir was fabricated in PDMS; the reservoir was bonded to the glass/electrodes using O_2_ plasma exposure. In each experiment a 50 μL aliquot of nanosphere suspension was pipetted into the PDMS reservoir on top of the device. A glass cover slip was then used to prevent sample evaporation.

Carboxylate‐modified 200nm diameter latex spheres (Invitrogen Molecular Probes, Eugene, OR) with yellow‐green fluorescence (505/515 nm wavelength) were washed three times in KCl electrolyte solution (2×10^−4^ S/m) and suspended in the same medium at a concentration of 5 × 10^10^ spheres/mL (diluted 1:100 from 2% w/v stock solution). The concentration and monodispersity of the nanospheres was verified (data not shown) using a NanoSight™ LM10 particle analyzer (NanoSight, Wiltshire, UK). The motion of the nanospheres was observed using an inverted microscope (Axiovert 200M, Zeiss, Germany) with epi‐fluorescent illumination (HBO100, Zeiss), imaged with ×40 objective, 0.75 numerical aperture, and recorded with a digital camera (DCC1240M, Thorlabs USB 2.0, Newton, NJ) with resolution 1280 by 1024 pixels per frame. Videos were analyzed using software written in Mathematica™ 8 (Wolfram Research, Champaign, IL) and Matlab™ 7.14 (Mathworks, Natick, MA). Nanosphere collections at the high‐field gradient regions of the castellated electrodes, evident in Fig. 1B(ii), were calibrated by measuring the fluorescence intensity at the electrode tips and using the same procedures as previously described 16.

## 3 Results

**Figure 4:**
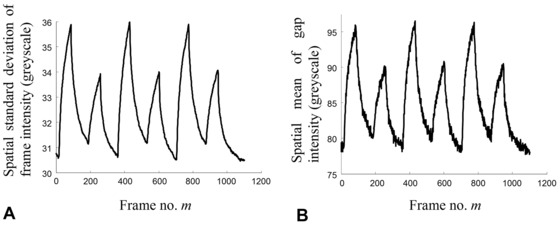
Dual‐cycle pDEP collection and release quantified using (A) spatial SD of frame intensity that is sensitive to the tail shown in Fig. 3B, and (B) spatial mean of gap intensity that is conventional to measure and requires knowledge of electrode geometry. Comparing (A) and (B) shows, apart from the scale, which does not affect the initial collection rate ratios, that the profiles are very similar. Since the frame SD is much easier to compute that the gap mean, it can be processed in real‐time concurrent with video acquisition.

A comparison of the spatial SD of frame intensity, $\sigma_{F}\left(m\right)$, and spatial mean of the interelectrode gap intensity, $\mu_{G}\left(m\right)$ for dual‐cycle continuously pulsed pDEP is shown in Fig. 4A and B, respectively.

The control and probe frequencies were $f_{1}\;\;=\;\;0.7$ MHz and $f_{2}\;\;=\;\;3.0$ MHz, respectively; all other parameters are as described in Section 2.3. The profiles show excellent correlation, that is, notwithstanding differences in their scale and baseline, their time profiles closely agree and concur with previous findings 2.

**Figure 5:**
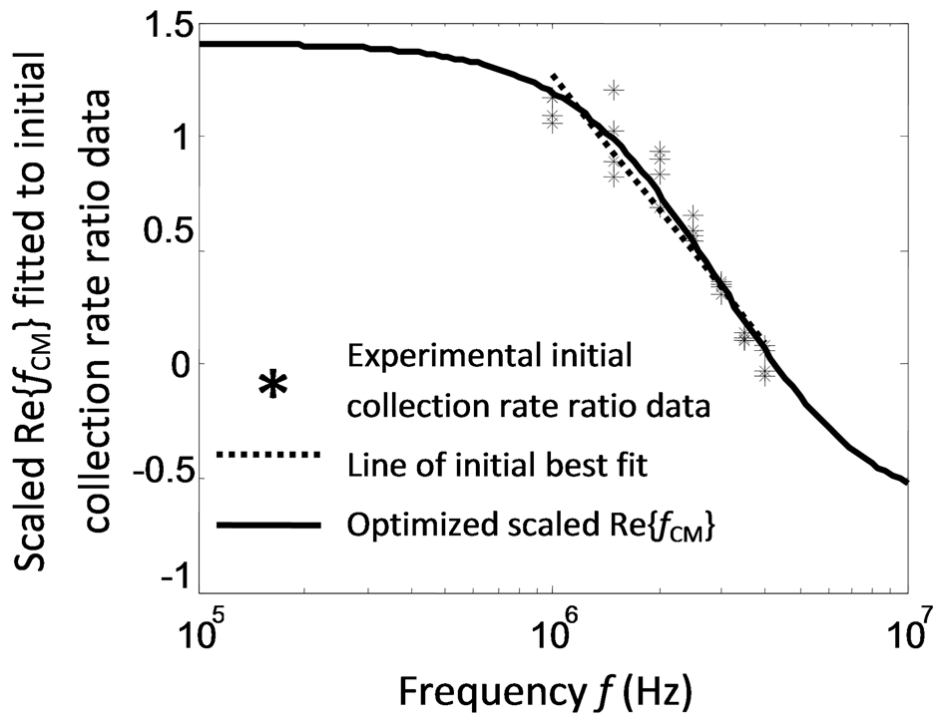
Dual‐cycle, on–off pulsed, frequency hopped pDEP initial collection rate ratio data as a function of frequency. The scaled real part of the Clausius–Mossotti function is fitted to data starting with a line of best fit and further refined. The fit yields values for the nanoparticle conductivity of 25.8 mS/m and surface conductance of 1.29 nS.

Referring to Fig. 2A and B, a further investigation used dual‐cycle, continuously pulsed DEP frequency collections with an “on” control pulse set at f=0.7 MHz and the probe frequencies spanning the range, f=1−4 MHz. Time‐dependent collection profiles for each of the control and probe cycles were quantified by linear fitting (fourteen data points) that approximates an exponential series for sufficiently short time intervals 5, 9, 10, 11, 12, 13, 14, 15, 16, 17, 18, 19, 20, 21, 22, 23. The initial collection rate ratio of the dual‐cycle control and probe, for each experiment, was evaluated using Eq. (9) in 1 or Supporting Information Eq. (S1.2). Representative data, ρ_i_, involving quadruple replicates for each of the seven applied frequencies (28 data values), was fitted to a scaled f CM R, with the resulting curve shown in Fig. 5. Joint estimates of *m*_s_ and σ_p_ were evaluated according to a two‐step, linear approximation, and Newton–Raphson algebraic and numerical refinement procedure derived and described in 1 and given by Supporting Information Eqs. (S2.1) to (S2.8). The first, initial, step involves a simple, “rough” fit to the collection rate ratio data and is shown by the straight line in Fig. 5. The second step refines, or optimizes the joint estimates of the first step, and is shown in Fig. 5 by the curve, msf CM R, fitted to the data. The plots in Fig. 5 are thus labeled, “Line of initial best fit” and “Optimized scaled Re{*f*_CM_}”, respectively. $f_{CM}^{R}$ values used in the calculation were $\sigma_{m}\;\;=\;\;0.2$ mS/m, ${ɛ}_{m}\;\;=\;\;78.4\;{ɛ}_{0}$ and ${ɛ}_{p}\;\;=\;\;2.55\;{ɛ}_{0}$ where ${ɛ}_{0}\;\;=\;\;8.8542\times 10^{-12}$ F/m is the permittivity of free space. The final joint estimates for the magnitude scaling factor and the nanoparticle conductivity were, $m_{s}\;\;=\;\;1.44$ and $\sigma_{p}\;\;=\;\;25.8$ mS/m, respectively. Assuming, as before, $\sigma_{b}\;\;=\;\;0$ and r=100 nm, then using the relation, $\sigma_{p}\;\;=\;\;\sigma_{b}\;\;+\;\;2K_{S}/r$ 1, 8, 26, 29, 30 or Supporting Information Eqs. (S1.3) and (S1.4), the estimate for the surface conductance was evaluated to be $K_{S}\;\;=\;\;1.29$ nS.

## 4 Discussion

In our pDEP experiments, the lower frequency range around 1 MHz and below is limited by the onset of fluid motion, particularly AC electroosmosis that tends to disrupt the pDEP collections 17, 37, 38, 39, 40, 41. To include the lower frequencies, therefore, would require including an AC electroosmosis model, at present a subject for future research and well beyond the scope of the current work. The upper frequency range of 4 MHz is limited by the onset of nDEP. A method for measuring dielectric parameters using nDEP could be developed along similar lines to pDEP. However, modifications in the procedures, or the use of three‐dimensional electrode traps, would be needed as nDEP of nanoparticles using planar arrays, often leads to diffusion into the bulk medium due to the random action of Brownian motion.

The spatial SD of the frame intensity, during the collection phases of the pulsed pDEP experiments, is shown in Fig. 4A and B to be proportional to the spatial mean of the interelectrode gap intensity, i.e. $\sigma_{F}\left(t\right)\;\;\propto \;\;\mu_{G}\left(t\right)$. The linear proportionality is mainly attributed to the bi‐model pixel distribution shown in Fig. 3B whereby the left “dark” mode, representing the electrode pixels, remains constant in time, while the tail of the right, “brighter” mode, representing the fluorescence across the gap, varies in accordance with pDEP nanoparticle collections. Consequently, time‐dependent pDEP driven shifts in the position of the mean of the gap pixel intensity, are detected by the frame SD that is sensitive to movements in the mode tail.

In this respect, the statistical method has been developed using an inverted microscope arrangement where the electrodes shadow the emitted fluorescence transmitted through the glass, thus, yielding a relatively stable left “dark” mode. It is not clear at this stage how successfully the method can be used with an upright epi‐fluorescent microscope that would capture the light reflected by the electrodes, although current indications are promising. The statistical method is suitable for black and white or color images and for a range of investigations involving intricate and extensive electrode designs that would be much more difficult with conventional geometrical filtering. As shown by Eq. (2), statistical filtering is *independent* of geometry, so that the method can work with castellated electrodes with other feature sizes, and other electrode designs, for example, interdigitated or polynomial. The statistical method has been demonstrated using 2D planar arrays with small 5 micron castellated features, shown in Fig. 1B, that we reported previously to require the development of specialized methods to achieve geometric‐based image quantification 2. Therefore, the statistical method avoids the need for resource‐consuming development and present indications are that it is not limited only to 2D planar electrodes; it could be used for 3D electrodes. Future work is planned to investigate the opportunities for insulating, or electrode‐less DEP, and identify the generality and limitations of the method.

The algorithmic and computational simplicity of statistical method, as evidenced by Eq. (2) enables rapid, real‐time, supervisor‐free image quantification; features that are useful to the experimentalist. Statistical measures, such as the SD are easy to implement, using software such as Matlab™, Mathematica™, or LabVIEW™ for live video processing, and requires little programming experience. We are not aware of any published supervisor‐free DEP image analysis methods, the closest being the very recent work of Rohani et al. 35. They have also developed a technique for reducing the noise in their image processing. By selecting a threshold pixel value they extract only portions of their image for which the DEP process is occurring. Although they have not implemented it for use with a complex electrode geometry, we are aware from our work examining different pixel intensity values, that their method could also be suitable to perform supervisor‐free RoI extraction from such complex geometries.

One drawback of their method is that, due to its nature, they are forced to select a threshold value; although they take the sensible option of determining the threshold determined by a fixed number of SDs from the mean pixel intensity, this requires a certain amount of arbitrary choice, and could introduce systematic error. Our approach uses information from the whole image and does not involve such a choice. Ultimately the two techniques are complimentary; while our method should be simpler and more robust for collection rate experiments with complex electrode geometries, it would be interesting to extend their method to investigate nanoparticle kinetics for different threshold pixel values.

The values for the nanoparticle conductivity, $\sigma_{p}\;\;=$ 25.8 mS/m, and surface conductance, $K_{S}\;\;=\;\;1.29$ nS, using the statistical method concur closely with earlier work that used the geometrical method 1, 15. Other experiments also showed agreement between the statistical and geometrical methods (data not shown). Statistical filtering, using the SD, therefore, yields accurate and reliable collection rate measurements that can be used for evaluating dielectric properties, such as the nanoparticle conductivity and surface conductance. A novel, robust, and easy‐to‐implement statistical filtering method is demonstrated for quantifying pDEP behavior using video images of nanoparticles. The method does *not* require any input about electrode array geometry and allows accurate, real‐time, robust, and supervisor‐free quantification of nanoparticle dynamics.

## Supporting information

As a service to our authors and readers, this journal provides supporting information supplied by the authors. Such materials are peer reviewed and may be re‐organized for online delivery, but are not copy‐edited or typeset. Technical support issues arising from supporting information (other than missing files) should be addressed to the authors.

Fig. S1. pDEP collection rate ratio data as a function of frequency. The data are fitted to a scaled starting with a line of best fit and refined with the scaled , as shown. The fit yields values for the nanoparticle conductivity of 25.8 mS/m and surface conductance of 1.29 nS.
