# Supplementary material for: Real‐time dielectrophoretic signaling and image quantification methods for evaluating electrokinetic properties of nanoparticles
Source: Electrophoresis. 2015 Jul 6;36(13):1443–50. doi: 10.1002/elps.201400500 (PMC5034756; doi:10.1002/elps.201400500)
Supplement: Supplementary file 1 — Fig. S1. pDEP collection rate ratio data as a function of frequency. The data are fitted to a scaled starting with a line of best fit and refined with the scaled , as shown. The fit yields values for the nanoparticle conductivity of 25.8 mS/m and surface conductance of 1.29 nS. [file ELPS-36-1443-s001.docx]

**Supporting Information**

**Real-time dielectrophoretic signaling and image quantification methods for evaluatingelectrokineticproperties of nanoparticles**

David J. Bakewell, Joe Baileyand David Holmes

NB: Please note that the numbered references are the same as those listed in the manuscript.

***S1: Clausius-Mossotti factor for a sphere and initial collection rate ratio***

Referring to Eq. (1) in the main article text, the real part of the Clausius-Mossotti (CM) function, for a spherical particle, can be written,

$\mathrm{Re}\{f_{CM}[\omega(t)]\}=\frac{(\sigma_{p}-\sigma_{m})(\sigma_{p}+2\sigma_{m}) +\omega(t)^{2}(\varepsilon_{p}-\varepsilon_{m}) (\varepsilon_{p}+2\varepsilon_{m})}{(\sigma_{p}+2\sigma_{m}) ^{2} + \omega(t)^{2}(\varepsilon_{p}+2\varepsilon_{m}) ^{2}} \equiv f_{CM}^{R}$ (S1.1)

where$\varepsilon_{p}$and $\sigma_{p}$are the permittivity and conductivity of the particle, and $\varepsilon_{m}$and$\sigma_{m}$ are the permittivity and conductivity of the medium. In (S1.1) the angular frequency is$\omega(t) = 2\pi f(t)$ where it is understood $f(t)$ is switched on and remains constant for a limited time (up to a minute) then switched off. On the right side of (S1.1) the symbol for the real part of the CM function is abbreviated, for convenience, as$f_{CM}^{R}$.

The ratio of the collection rates, $\rho$, expressed by Eq. (9) in [1], assumes that the experimental parameters for nanoparticle collection phases remain the same *except* for the probe frequency. The assumption is valid for experiments in this paper, so that the ratio, for the *i*th probe frequency, can be written as

(S1.2)

where$n$is the nanoparticle number within a designated 3D volume typically including the electrode edges, e.g. [15-17], is the rate of change of $n$with respect to time (denoted by the overhead dot)and it is understood$t$denotes the time point at the start of each collection phase. The subscripts ‘0’, and the subscript index $i = 1, 2, 3 ...$denote control and probe phases, respectively. That is, referring to Fig. 2A, the first cycle collection phase uses a constant frequency that acts as a *control*, i.e.$f(t) = f_{0}$, and the second cycle collection phase uses a frequency that is made variable so it can *probe* the DEP response for a range of selected frequencies, i.e.$f(t) = f_{i}, i = 1, 2, 3, ....$.

On the right side of (S1.2), $m_{s}$ denotes a $f_{CM}^{R}$ *magnitude scaling* factor,$m_{s}=1/\mathrm{Re}\{f_{CM}[\omega_{0}(t_{0})]\}$ where typically $1 < m_{s} < 2$and remains constant since the control frequency remains *fixed*. Consequently, $\rho$ is essentially a scaled $f_{CM}^{R}$ and it is *no* longer bounded between -0.5 and 1. Conceptually, it is important to note that the scaled $f_{CM}^{R}$in (S1.2) involves at least *six* parameter values to yield a single value of the ratio,$\rho_{i}$, i.e.$\varepsilon_{m}$, $\sigma_{m}$, $\varepsilon_{p}$, $\sigma_{p}$, $m_{s}$ and $\omega_{ i}$.

Since six parameters are needed to yield a single value, $\rho_{i}$ in (S1.2), it follows that for a 1:1 mapping, which is applicable in this case, an *inverse* process involving a different set of six parameters, namely,$\varepsilon_{m}$, $\sigma_{m}$, $\varepsilon_{p}$, $m_{s}$, $\omega_{ i}$and $\rho_{i}$ can be used to determine$\sigma_{p}$. In practice, due to experimental randomness, a number of initial collection rate ratio samples, $\rho_{i}$, with their corresponding probe frequencies, $\omega_{ i}$, are needed,i.e. $\{\rho_{ i}\} = \{\rho_{ 1}, \rho_{ 2}, \rho_{ 3}, ....\}$ and $\{\omega_{ i}\} = \{\omega_{ 1}, \omega_{ 2}, \omega_{ 3}, ....\}$. Three of the dielectric parameters,$\varepsilon_{m}$, $\sigma_{m}$, and $\varepsilon_{p}$,and values for the independent variable $\{\omega_{ i}\}$are known, and $\{\rho_{i}\}$ are measured. Hence, fitting samples of ratios, $\{\rho_{i}\}$ to a scaled$f_{CM}^{R}$ for a range of frequencies, yields jointexperimental data based estimates for$m_{s}$ and$\sigma_{p}$, as described in [1] and in section 4 of the main article text. Using the relation[1, 8, 26, 29, 30],

$\sigma_{p} = \sigma_{b} + 2K_{S}/r$ (S1.3)

where the conductivity of the nanoparticle bulk latex, $\sigma_{b} = 0$ and the nanoparticle radius, $r$, is known. Rearranging (S1.3) and substituting,

$K_{S} = \sigma_{p} r/2$ (S1.4)

enables experimental data-based estimation of the surface conductance, $K_{S}$.

***S2: Estimation of nanoparticle parameters by fitting the Clausius-Mossotti function to collection rate data***

The proportional relationship between the real part of CM, that is bounded, $-1/2 \leq f_{CM}^{R} \leq1$ and the unbounded collection rate ratio, *ρ*, expressed by (S1.2), suggests that they should be related by a scaling factor, $m_{s}$, and constant *c* such that

$\rho= m_{s} .f_{CM}^{R}+c$ (S2.1).

where, as before, $1 < m_{s} < 2$. A feature of (S1.2), verified experimentally, is that if $f_{CM}^{R} =0$ at cross-over then$\rho=0\Rightarrow c=0$. The fitting process, therefore, entails estimating from collection ratio data two parameters: (1) nanoparticle conductivity, $\sigma_{p}$, and (2) scaling factor, $m_{s}$. Since (1) and (2) are independent, the fitting process is two dimensional and entails *two-steps* jointly performed:

Step (1) *initiation* – where an *initial* values for $\sigma_{p}$ and $m_{s}$are estimated from collection rate data, followed by

Step (2) *refinement*– where initial estimates of $\sigma_{p}$ and $m_{s}$are *refined* by fitting the nonlinear function, $f_{CM}^{R}(f)$ using Newton-Raphson method or a numerical method, e.g. Nelder-Mead.

Step 1: finds initial, rough estimates for equation parameters of the line of best fit shown in Fig. 5 that relates the initial collection rate ratio, *ρ*, with log-frequency, *l*,

$\rho^{lin}=\alpha+\beta l$ (S2.2)

where the abscissa, *α*, and gradient, *β*, are given by standard textbook formulae, e.g. [36], for least sum of squared error (SSE) fit, see Appendix A attached. From Eq. (SA. 4)

$\hat{\beta}=\frac{s_{n}\sum_{i=1}^{s_{n}} \rho_{i}l_{i}-\sum_{i=1}^{s_{n}} \rho_{i}\sum_{i=1}^{s_{n}} l_{i}}{s_{n}\sum_{i=1}^{s_{n}} l_{i}^{2}-\left(\sum_{i=1}^{s_{n}} l_{i}\right)^{2}}$ (S2.3)

and from Eq. (SA.2),

$\hat{\alpha}=\frac{1}{s_{n}}\sum_{i=1}^{s_{n}} \rho_{i}-\frac{\beta}{s_{n}}\sum_{i=1}^{s_{n}} l_{i}= \bar{\rho}-\beta\bar{l}$ (S2.4).

An initial estimate for the scaling factor can be given by the reciprocal of the root mean sum-of-squares given by Eq. (31) in [1] applicable for a *set* of initial collection rate ratio measurements $\{\rho_{i}\}$,

$m_{0}=\left(\frac{1}{s_{n}}\sum_{i=1}^{s_{n}} \rho_{i}^{2}\right)^{-1/2}$ (S2.5)

where the subscript ‘s’ is replaced by the subscript ‘0’ that denotes ‘initial estimate’ and $s_{n}$ is the number of samples (data points); referring to Fig. 5, $s_{n} = 4\times7 = 28$. The estimate for the cross-over frequency line-of-best fit, or if it is not measured, extrapolation of the line, is found by setting $\rho^{lin}= 0$ in (S2.2) so that the log-frequency, $l = -\alpha/\beta$ yields an estimate, $\hat{f}_{x}= 10^{-\alpha/\beta}$.

The initial estimate for the nanoparticle conductivity is then determined from Eq. (SA.7) in Appendix A,

$\sigma_{p}=\frac{-\sigma_{m}+[9 \sigma_{m}^{2}-4 \omega_{x}^{2} (\varepsilon_{p}-\varepsilon_{m})(\varepsilon_{p}+2\varepsilon_{m})]^{1/2}}{2}$ (S2.6)

where $\sigma_{m} = 0.2$mS/m, $\varepsilon_{m} = 78.4\varepsilon_{0}$, $\varepsilon_{p} = 2.55\varepsilon_{0}$ and $\varepsilon_{0} = 8.8542\times10^{-12}$ F/m is the permittivity of free space. In (S2.6) where the subscript ‘p’ is can be replaced by the subscript ‘0’ to denote ‘initial’.

In the example shown in Fig. 5 and Fig. S1 below, the estimates for the line of best fit, etc., from the relations given above are $\alpha\cong13.0, \beta\cong-1.95$ and$\hat{f}_{x}\cong4.46$ MHz. The line of best fit is shown in Fig. 5 and Fig. S1 below. The estimates in turn, lead to initial estimates $\hat{m}_{0}= 1.43$ and $\hat{\sigma}_{0} = 27.2$ mS/m in determining the scaled real part of the CM function, $\hat{m}_{0}\mathrm{Re}\{f_{CM}(f,\hat{\sigma}_{0})\}$ labeled as ‘Initial scaled Re{*f*_CM_}’ in Fig. S1 below (for clarity not shown in Fig. 5). Note that only a few significant figures are quoted above, the evaluation in Matlab used high precision. The fitted curve is close to the data collection ratios but needs further refinement.

Step 2: The initial estimate of nanoparticle conductivity $\hat{\sigma}_{0}$ is refined by updating using the Newton-Raphson (NR) procedure, i.e. $\hat{\sigma}_{0}$ is updated to $\hat{\sigma}_{1}$ by the algorithm given by Eq. (SA.15) in Appendix A

$\sigma_{k+1}=\sigma_{k}-\frac{\partial_{\sigma_{k}}SSE}{\partial_{\sigma_{k}, \sigma_{k}}SSE}$ (S2.7)

where it is understood for the first iteration (in this example) *k* = 0 and $\sigma_{k} \equiv\hat{\sigma}_{p}$ for the *k*^th^ iteration, $k = 0, 1, 2, 3 , ....$. In (S2.7), the first and second partial derivatives of the SSE, involving $f_{CM}^{R}(f)$are evaluated using the expressions given by Eq. (SA.11) to Eq. (SA.14) in Appendix A. The revised conductivity,$\hat{\sigma}_{k + 1}$, then enables estimate for the scaling factor, $\hat{m}_{k}$, to be updated to $\hat{m}_{k+1}$. It is given by Eq. (SA.10) in Appendix A

$\hat{m}_{k+1}=\sum_{i=1}^{s_{n}} \rho_{i}f_{CM}^{R}(l_{i},\sigma_{k+1})/\sum_{i=1}^{s_{n}} [f_{CM}^{R}(l_{i},\sigma_{k+1})]^{2}$ (S2.8).

The iteration is repeated until the difference is sufficiently small, e.g. $\sigma_{k+1} -\sigma_{k} < 0.01$mS/m. For the collection ratios shown in Fig. 5 and Fig. S1, the refined fit, $\hat{m}_{s}\mathrm{Re}\{f_{CM}(f,\hat{\sigma}_{p})\}$ is called the ‘Optimized scaled Re{*f*_CM_}’. It is a better fit to the collection ratio values than the initial fit shown. The final joint estimates for the magnitude scaling factor and the nanoparticle conductivity, after about a dozen iterations for this example, were $m_{s} = 1.44$ and $\sigma_{p} = 25.8$mS/m, respectively.

10

5

10

6

10

7

-1

-0.5

0

0.5

1

1.5

Scaled Re{*f*_CM_} fitted to

initial collection rate ratio data

Frequency *f* (Hz)

Experimental initial collection rate ratio data

Line of initial best fit

Initial scaled Re{*f*_CM_}

Optimized scaled Re{*f*_CM_}

*

Fig. S1. pDEP collection rate ratio data as a function of frequency. The data are fitted to a scaled $f_{CM}^{R}$ starting with a line of best fit and refined with the scaled $f_{CM}^{R}$, as shown. The fit yields values for the nanoparticle conductivity of 25.8 mS/m and surface conductance of 1.29 nS.

**Supplementary Information: Appendix A.**

*Step 1: Expressions for estimate initiation:*

An initial estimate for nanoparticle conductivity uses the line of best fit. This can be derived by taking the sum of the squares of the residual error, $e_{i}$,(SSE) between the collection rate ratio data, $\rho_{i}$, and the line to be fitted,

$SSE=\sum_{i=1}^{s_{n}} e_{i}^{2}=\sum_{i=1}^{s_{n}} [\rho_{i}-(\beta l_{i} + \alpha)]^{2}$ (SA.1).

The abscissa, *α*, is found by finding the minimum SSE, i.e. setting the partial derivative of SSE, with respect to that parameter, to zero, $\partial SSE/\partial\alpha=0$ and yields the estimate (denoted by ‘^’ overhead),

$\hat{\alpha}=\frac{1}{s_{n}}\sum_{i=1}^{s_{n}} \rho_{i}-\frac{\beta}{s_{n}}\sum_{i=1}^{s_{n}} l_{i}= \bar{\rho}-\beta\bar{l}$ (SA.2).

Similarly, the gradient, *β*, is found by finding the minimum SSE, setting the partial derivative to zero,

$\frac{\partial SSE}{\partial\beta}=-2\sum_{i=1}^{s_{n}} [\rho_{i}-(\beta l_{i} + \alpha)]l_{i}=0$ (SA.3)

and leads to the standard textbook formula for least squares fit,

$\hat{\beta}=\frac{s_{n}\sum_{i=1}^{s_{n}} \rho_{i}l_{i}-\sum_{i=1}^{s_{n}} \rho_{i}\sum_{i=1}^{s_{n}} l_{i}}{s_{n}\sum_{i=1}^{s_{n}} l_{i}^{2}-\left(\sum_{i=1}^{s_{n}} l_{i}\right)^{2}}$ (SA.4).

If the real part of the CM factor is assigned a single real, bounded value,$-1/2 < \kappa< 1$ and for convenience and clarity, the frequency assumed constant, $\omega(t) = \omega= 2\pi f$ then Eq. (S1.1) becomes the standard expression for the$f_{CM}^{R}$,

$\begin{matrix} \mathrm{Re}\{f_{CM}(\omega)\} = \mathrm{Re}\left\{\frac{\underline{\varepsilon}_{p}^{*}-\underline{\varepsilon}_{m}^{*}}{\underline{\varepsilon}_{p}^{*}+2\underline{\varepsilon}_{m}^{*}}\right\} \\ =\frac{(\sigma_{p}-\sigma_{m})(\sigma_{p}+2\sigma_{m}) +\omega^{2}(\varepsilon_{p}-\varepsilon_{m}) (\varepsilon_{p}+2\varepsilon_{m})}{(\sigma_{p}+2\sigma_{m}) ^{2} + \omega^{2}(\varepsilon_{p}+2\varepsilon_{m}) ^{2}}\equiv\kappa\end{matrix}$ (SA.5).

Re-arranging into a quadratic dependence yields a solution with one positive real root

$\sigma_{p}=\frac{-\sigma_{m}(1-4\kappa)+[\sigma_{m}^{2}(1-4\kappa)^{2}-4(1-\kappa)g]^{1/2}}{2(1-\kappa)}$ (SA. 6)

where $g=\omega^{2}[\varepsilon_{p}^{2}(1-\kappa)+\varepsilon_{p}\varepsilon_{m}(1-4\kappa)-2\varepsilon_{m}^{2}(1+2\kappa)]-2\sigma_{m}^{2}(1+2\kappa)$ . Imposing the joint condition $\kappa= 0$ at the cross-over frequency, $\omega= \omega_{x}$, and re-arranging,

$\sigma_{p}=\frac{-\sigma_{m}+[9 \sigma_{m}^{2}-4 \omega_{x}^{2} (\varepsilon_{p}-\varepsilon_{m})(\varepsilon_{p}+2\varepsilon_{m})]^{1/2}}{2}$ (SA.7)

yields the expression given by Eq. (18) in [1] that also concurs with the literature, e.g. [8].

*Step 2: Expressions for estimating refinement*:

The SSE between the ratios of collection rate experimental data, $\rho_{i}$, and data predicted from the product of the scaling factor, $m_{s}$ and $f_{CM}^{R}$for the *i*th sample at the log-frequency, $l_{i}=\log_{10}f_{i}$, can be written as,

$SSE=\sum_{i=1}^{s_{n}} e_{i}^{2}=\sum_{i=1}^{s_{n}} [\rho_{i}-m_{s}f_{CM}^{R}(l_{i})]^{2}$ (SA.8).

The optimal value for the scaling factor is found by finding the partial derivative of SSE with respect to that parameter and setting to zero to find the minimum,

$\frac{\partial SSE}{\partial m_{s}}=-2\sum_{i=1}^{s_{n}} [\rho_{i}-m_{s}f_{CM}^{R}(l_{i})]f_{CM}^{R}(l_{i})=0$ (SA.9)

which leads to

$\hat{m}_{s}=\sum_{i=1}^{s_{n}} \rho_{i}f_{CM}^{R}(l_{i})/\sum_{i=1}^{s_{n}} [f_{CM}^{R}(l_{i})]^{2}$ (SA.10).

Minimizing SSE with respect to the nanoparticle conductivity, the first and second partial derivatives are

$\partial_{\sigma_{k}}SSE=\frac{\partial SSE}{\partial\sigma_{k}}=2\sum_{i=1}^{s_{n}} \rho_{i}. \partial_{\sigma_{k}}f_{i}^{R}-2m_{s}\sum_{i=1}^{s_{n}} f_{i}^{R}. \partial_{\sigma_{k}}f_{i}^{R}$ (SA.11)

and

$\partial_{\sigma_{k}, \sigma_{k}}SSE=\frac{\partial^{2}SSE}{\partial\sigma_{k}^{2}}=2\sum_{i=1}^{s_{n}} \rho_{i}. \partial_{\sigma_{k},\sigma_{k}}f_{i}^{R}-2m_{s}\sum_{i=1}^{s_{n}} [f_{i}^{R}. \partial_{\sigma_{k},\sigma_{k}}f_{i}^{R} + (\partial_{\sigma_{k}}f_{i}^{R})^{2}]$ (SA.12)

where standard notation is understood $f_{i}^{R}\equiv f_{CM}^{R}(l_{i})$, $\partial_{\sigma_{k}}f_{i}^{R}\equiv\frac{\partial f_{CM}^{R}(l_{i})}{\partial\sigma_{k}}$ and $\partial_{\sigma_{k}, \sigma_{k}}f_{i}^{R}\equiv\frac{\partial^{2}f_{CM}^{R}(l_{i})}{\partial\sigma_{k}^{2}}$ for the *k*th iteration of the conductivity estimate. Explicit forms for the partial derivatives of $f_{CM}^{R}$, after some algebra, are given by

$\partial_{\sigma_{p}}f_{i}^{R}\equiv\frac{\partial f_{CM}^{R}}{\partial\sigma_{p}}= \frac{3 \sigma_{m} \sigma_{p}^{2}+a\sigma_{p}+b}{[(\sigma_{p}+2\sigma_{m}) ^{2} + \omega^{2}(\varepsilon_{p}+2\varepsilon_{m}) ^{2}]^{2}}$ (SA.13)

and

$\partial_{\sigma_{p}, \sigma_{p}}f_{i}^{R}\equiv\frac{\partial^{2}f_{CM}^{R}}{\partial\sigma_{p}^{2}}= \frac{-6 \sigma_{m} \sigma_{p}^{3}+-3a\sigma_{p}^{2}+c\sigma_{p}+d}{[(\sigma_{p}+2\sigma_{m}) ^{2} + \omega^{2}(\varepsilon_{p}+2\varepsilon_{m}) ^{2}]^{3}}$ (SA.14)

where, in (SA.10) and (SA.11), for pedagogical purposes, the *k*th iteration index has been set for convenience, $'k' \equiv'p'$. The coefficients in (SA.13) and (SA.14) are $a = 6 [2\sigma_{m}^{2} + (\sigma_{p}+2\varepsilon_{m})\varepsilon_{m}\omega^{2}]$, $b = 3\varepsilon_{m} [4\sigma_{m}^{2} + (4\varepsilon_{m}^{2}-\varepsilon_{p}^{2}) \omega^{2}]$, $c = 18\sigma_{m} [-4\sigma_{m}^{2} + (\varepsilon_{p}^{2}-4\varepsilon_{m}^{2}) \omega^{2}]$ and $d = 6\omega^{2}(\varepsilon_{p}+2\varepsilon_{m}) [6\varepsilon_{p}\sigma_{m}^{2} + \varepsilon_{m}(\varepsilon_{p}+2\varepsilon_{m})^{2} \omega^{2}]-48\sigma_{m}^{4}$. Eq. (SA.13) and (SA.14) are then substituted into (SA.11) and (SA.12), and thence into the Newton-Raphson method for solving a nonlinear equation,

$\sigma_{k+1} = \sigma_{k} -\frac{\partial_{\sigma_{k}}SSE}{\partial_{\sigma_{k}, \sigma_{k}}SSE}$ (SA.15).

------------
